# High-Sensitivity Cardiac Troponin Concentrations in Patients with Chest Discomfort: Is It the Heart or the Kidneys As Well?

**DOI:** 10.1371/journal.pone.0153300

**Published:** 2016-04-20

**Authors:** Eline P. M. Cardinaels, Sibel Altintas, Mathijs O. Versteylen, Ivo A. Joosen, Laurens-Jan C. Jellema, Joachim E. Wildberger, Marco Das, Harry J. Crijns, Otto Bekers, Marja P. van Dieijen-Visser, Bastiaan L. Kietselaer, Alma M. A. Mingels

**Affiliations:** 1 Central Diagnostic Laboratory, Department of Clinical Chemistry, Cardiovascular Research Institute Maastricht (CARIM), Maastricht University Medical Center (MUMC+), Maastricht, the Netherlands; 2 Department of Cardiology, CARIM, MUMC+, Maastricht, the Netherlands; 3 Department of Clinical Chemistry and Hematology, Gelre Hospitals, Apeldoorn, the Netherlands; 4 Department of Radiology, CARIM, MUMC+, Maastricht, the Netherlands; University of Colorado Denver, UNITED STATES

## Abstract

**Background:**

High-sensitivity cardiac troponins (hs-cTn) are the preferred biomarkers to detect myocardial injury, making them promising risk-stratifying tools for patients with symptoms of chest pain. However, circulating hs-cTn are also elevated in other conditions like renal dysfunction, complicating appropriate interpretation of low-level hs-cTn concentrations.

**Methods:**

A cross-sectional analysis was performed in 1864 patients with symptoms of chest discomfort from the cardiology outpatient department who underwent cardiac computed tomographic angiography (CCTA). Serum samples were analyzed using hs-cTnT and hs-cTnI assays. Renal function was measured by the estimated glomerular filtration rate (eGFR), established from serum creatinine and cystatin C. On follow-up, the incidence of adverse events was assessed.

**Results:**

Median hs-cTnT and hs-cTnI concentrations were 7.2(5.8–9.2) ng/L and 2.6(1.8–4.1) ng/L, respectively. Multivariable regression analysis revealed that both assay results were more strongly associated with eGFR (hs-cTnT:stβ:-0.290;hs-cTnI:stβ:-0.222) than with cardiac imaging parameters, such as coronary calcium score, CCTA plaque severity score and left ventricular mass (all p<0.01). Furthermore, survival analysis indicated lower relative risks in patients with normal compared to reduced renal function for hs-cTnT [HR(95%CI), 1.02(1.00–1.03) compared to 1.07(1.05–1.09)] and hs-cTnI [1.01(1.00–1.01) compared to 1.02(1.01–1.02)] (all p<0.001).

**Conclusion:**

In patients with chest discomfort, we identified an independent influence of renal function on hs-cTn concentrations besides CAD, that affected the association of hs-cTn concentrations with adverse events. Estimating renal function is therefore warranted when interpreting baseline hs-cTn concentrations.

## Introduction

Identifying chest pain patients at risk for cardiovascular events remains an ongoing challenge [1]. A promising and cost-effective way to identify those “vulnerable” patients is the use of cardiac troponins [2,3]. Because of their unique cardiospecificity, cardiac troponins T (cTnT) or I (cTnI) are considered the preferred biochemical markers to detect myocardial injury and to diagnose acute myocardial infarction (AMI) in particular [4]. Since the introduction of high-sensitivity cardiac troponin (hs-cTn) assays, more accurate detection of low levels of circulating cardiac troponins became feasible [5], which significantly improved the diagnostic performance in patients with acute cardiac risk [6]. Even below the diagnostic cut-off, hs-cTn concentrations turned out to have an important prognostic value for acute cardiovascular events [7–9]. Moreover, in patients with stable coronary artery disease (CAD) low concentrations of hs-cTnT have been associated to the extent of CAD [2] and coronary plaque phenotypes that are more prone to rupture [3].

Unfortunately, the shift to more sensitive assays is accompanied by a reduction in specificity, as circulating hs-cTn levels are elevated in many other conditions besides AMI [7,10]. Renal dysfunction is one of those conditions in which elevated cardiac troponin concentrations are commonly detected [11,12]. Recently it was shown in chronic kidney disease (CKD) patients that elevated hs-cTn concentrations are indeed associated with reduced renal function [13]. Therefore, the interpretation of baseline hs-cTn values in the individual patient is complicated not only by cardiac disease [14–16] but also renal dysfunction.

As of yet it is unknown to what extent renal function contributes to higher cardiac troponin concentrations in stable patients with chest discomfort, in whom circulating hs-cTnT concentrations are mainly attributed to the presence and severity of atherosclerotic plaques [2,3] or echocardiographic abnormalities [17–19]. In-depth understanding in which way renal function affects hs-cTnT and hs-cTnI concentrations is of utmost importance for the use of hs-cTn values in clinical practice.

This study is the first to evaluate the impact of a decreased renal function on both hs-cTnT and hs-cTnI concentrations relative to the presence of cardiovascular disease in patients who visited the cardiology outpatient department with symptoms of chest discomfort. Moreover, the renal influence on the association of hs-cTn with the incidence of adverse events is investigated.

## Materials and Methods

### Study cohort

This study was approved by the Institutional Review Board (IRB) and Ethics Committee at the Maastricht University. Involved data were collected on a routine basis within the Maastricht Biomarker CT study (ClinicalTrials.gov NCT01671930, MEC 08-4-057) and data were analyzed anonymously in accordance with IRB guidelines. The study complies with the ethical principles of the Helsinki Declaration.

**Fig 1:**
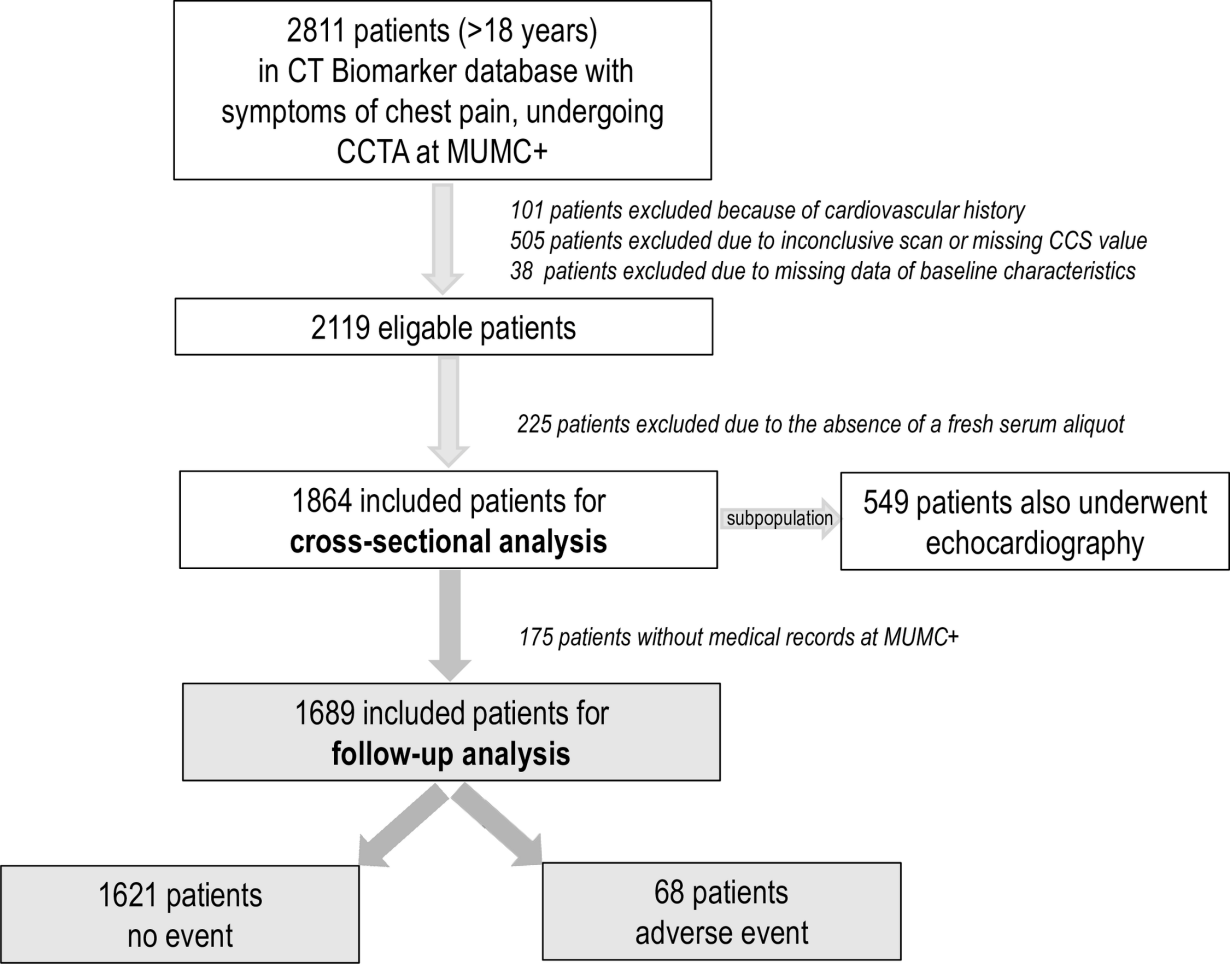
Flow chart of included and excluded patients.

We analyzed a cohort of 1864 consecutive patients who were enrolled in the Maastricht Biomarker CT Study. This cohort is comprised of patients from the cardiology outpatient department presenting with (a)typical chest pain with a low-to-intermediate pretest probability who were referred for CCTA for the evaluation of stable coronary artery disease (CAD), in accordance with the current guidelines [1,20]. Included were patients of whom serum was collected prior to CCTA and excluded were patients with a previous history or diagnosis of ACS at the time of CCTA and patients with severe renal dysfunction or on dialysis (due to application of contrast fluids) (Fig 1).

Previous results from the Maastricht Biomarker CT Study and additional specifications of this population have been published elsewhere [2,21,22].

### Biochemical analysis

Serum samples were collected immediately before CCTA, processed within 2 hours and directly stored at -80°C until analysis. Total cholesterol, triglycerides, high-density and low-density lipoprotein concentrations were measured as previously described [2]. Serum creatinine, cystatin C and cTnT concentrations were measured on the Cobas 6000 analyzer (Roche Diagnostics) in a fresh aliquot. Creatinine concentrations were assessed using the enzymatic method (Roche). Cystatin C was measured using a new particle-enhanced turbidimetric assay (Gentian AS), that was standardized against the certified ERM-DA471/IFCC cystatin C reference material [23]. The glomerular filtration rate was estimated using the Chronic Kidney Disease Epidemiology Collaboration equations [24] using serum creatinine and cystatin C concentrations. cTnT concentrations were determined using the high-sensitivity cTnT assay (Roche; lotnumber 167650), with a 99^th^ percentile upper reference limit of 14 ng/L and a 10% coefficient of variation (CV) cut-off at 13 ng/L. Gender-specific cutoffs were reported at 14.5 ng/L and 10 ng/L for males and females, respectively [5]. cTnI measurements were performed on the ARCHITECT i2000SR platform using the precommercial ARCHITECT STAT high-sensitivity troponin I (hs-cTnI) assay (Abbott Laboratories). According to the manufacturer, a 10% CV was reached at 4.7 ng/L and the 99^th^ percentile cut-off concentration at 26.2 ng/L for the overall population. Gender-specific cut-offs at 34.2 ng/L and 15.6 ng/L were also defined for males and females, respectively. In duplo measurements of multiple serum samples (>20) ranging between 7–12 ng/L in hs-cTnT-concentration and 3–4 ng/L in hs-cTnI-concentration, were measured with CVs of 3% and 9% respectively.

### Cardiac computed tomographic angiography

CCTAs were performed from December 2007 through December 2012 and analyzed as previously described [21]. In brief, CCTA were analyzed by two experts who were blinded from hs-cTn results. The coronary calcium score (CCS) was quantified by the Agatston method [25] and luminal plaque severity as assessed by CCTA was scored as no, mild (<50% stenosis), moderate (50–70% stenosis) and severe (>70% stenosis) CAD.

### Echocardiography

Echocardiography was performed in a subset of 549 patients (31%) within a period of 3 months from the CCTA scan by an expert echocardiographist, who was blinded for hs-cTn concentrations. Transthoracic images of the left ventricle (LV) were acquired to assess morphology, function and mass (Philips IE 33, Philips Medical Systems). LV function and -mass were calculated by off-line image analysis using Xcelera software package (Philips Medical Systems), according to current ESC/AHA guidelines [26]. Left ventricular hypertrophy was defined as an LVmass >95 g/m^2^ in females or LVmass >115 g/m² in males [27].

### Study endpoints

Electronic patient records were monitored for the occurrence of adverse events by two reviewers. Survival time was defined as the period from date of CCTA to date of the first event or the end of follow-up (June 2013). The composite study endpoints were defined in advance as mortality and major adverse cardiovascular events, encompassing acute coronary syndromes including AMI and unstable angina requiring hospitalization; and late coronary revascularization (>90 days after CCTA), such as percutaneous coronary interventions and coronary artery bypass grafting [22]. We cannot completely rule out that CCTA outcomes presented in this study were used in the decision to perform coronary revascularization in these patients during follow up. However, we corrected for this bias by excluding the procedures that were performed within 90 days after CCTA. No records could be retrieved of 175 subjects, therefore 1689 patients (91%) of the total cohort were included for follow-up analysis.

### Statistical analysis

Differences in baseline characteristics across hs-cTn categories were performed using the T-test for continuous variables with a normal distribution, Mann-Whitney U-test for non-normal distributed continuous variables and Chi square test for categorical variables. Data are presented as proportions, means ± standard deviations, and data with a non-normal distribution are given as the median (interquartile range, IQR). Pearson R correlation factors were calculated with the natural logarithm (Ln) of hs-cTnT and hs-cTnI, to normalize their skewed distribution. To assess the independent association of renal and cardiovascular parameters with hs-cTn concentrations, linear regression analyses were performed with either Ln(hs-cTnT) or Ln(hs-cTnI) as the dependent variable. Only the cardiovascular risk factors that were significantly associated to higher hs-cTnT concentrations were entered as independent variables into the multivariable model. R² was calculated to measure the performance of the model, and the R² change to address the additive effect of eGFR to the model. Interaction terms between eGFR and either CCTA and echocardiography-parameters were not statistically significant (p>0.05). Univariable and multivariable cox-proportional hazards models were performed to investigate the relationship of hs-cTn and the risk on adverse events during follow-up. Results are presented as hazard ratio (HR) and 95% confidence intervals (95%CI). None of the attending clinicians had access to the hs-cTnT and hs-cTnI concentrations, measured at the time of CCTA, for the diagnosis of outcome events. Statistical analysis was performed with SPSS 20.0 (SPSS). Two sided p-values of ≤0.05 were considered statistically significant.

## Results

### Distribution and determinants of hs-cTnT and hs-cTnI

Median (IQR) hs-cTn concentrations in this cohort were 7.2 (5.8–9.2) ng/L for hs-cTnT and 2.6 (1.8–4.1) ng/L for hs-cTnI. Of all patients, 6.6% (n = 123) were above the cutoff of hs-cTnT (14 ng/L) and 2.1% (n = 30) above the cutoff of hs-cTnI (26.2 ng/L). Using simple linear regression, we found a strong correlation between both hs-cTn concentrations (Pearson R 0.635, p<0.001). However, the biological equivalent for hs-cTnI to a hs-cTnT concentration of 14 ng/L was found to be at 6.4 ng/L (S1 Fig), which is in line with recently published results [28]. The majority of all patients (72%) had a normal renal function (eGFR>90 mL/min/1.73m²) and 98% had an eGFR above 60 mL/min/1.73m². The baseline characteristics are presented in Table 1 and illustrate that increasing quartiles of hs-cTnT and hs-cTnI were highly associated with traditional cardiovascular risk factors such as advancing age, male sex and increased blood pressure.

**Table 1:** Baseline characteristics in the overall population and by median hs-cTnT and hs-cTnI concentrations.

		hs-cTnT			hs-cTnI		
Determinant	All patients	≤7.2 ng/L	>7.2 ng/L	P-value	≤2.6 ng/L	>2.6 ng/L	P-value
**Traditional cardiovascular risk factors**							
Age,years	55.8±11.0	51.8±9.9	60.4±10.2	<0.001	53.0±10.7	58.1±10.6	<0.001
Males,%	56.0	41.3	69.2	<0.001	42.7	62.6	<0.001
Body mass index,kg/m²	27.0±4.4	26.6±4.3	27.3±4.3	<0.001	26.3±4.4	27.3±4.3	<0.001
Smokers,%	22.6	24.5	21.0	0.076	23.3	22.3	0.639
Diabetes,%	7.3	5.6	8.9	0.008	6.9	7.6	0.639
Family history,%	38.8	45.1	33.0	<0.001	43.6	36.3	0.002
Systolic blood pressure,mmHg	142.2±19.3	139.1±19.0	145.3±19.2	<0.001	138.1±18.2	144.5±19.6	<0.001
Diastolic blood pressure,mmHg	80.2±11.3	79.2±10.6	80.8±12.0	<0.001	78.3±10.8	80.9±11.6	<0.001
Total cholesterol,mmol/L	5.4±1.2	5.5±1.1	5.3±1.2	0.024	5.4±1.1	5.5±1.2	0.007
High density lipoprotein,mmol/L	1.3±0.5	1.3±0.4	1.3±0.5	0.001	1.4±0.4	1.3±0.5	0.001
Low density lipoprotein*,mmol/L	3.4±1.0	3.4±1.0	3.3±1.1	0.040	3.3±1.0	3.4±1.1	0.018
Triglycerides*,mmol/L	1.8±1.2	1.7±1.1	1.8±1.3	0.074	1.6±1.0	1.9±1.2	0.002
**Estimated GFR**							
CKD EPI _Creat-CysC_,mL/min/1.73m²	99.4±18.4	105.7±16.2	92.1±18.7	<0.001	105.1±17.7	95.2±18.5	<0.001
**CCTA**							
Coronary calcium score,AS	4.0(105.4)	0.0(0.0–25.3)	37.5(0.0–219.1)	<0.001	0.0(0.0–38.2)	16.3(0.0–167.7)	<0.001
Moderate-to-severe plaque,%	23.7	14.3	32.1	<0.001	17.4	26.7	<0.001

*, indicates 250 missing values

AS, indicates Agatston score.

### Independent association of cardiovascular disease and renal function with hs-cTn concentrations

Univariable regression analysis demonstrated that hs-cTn concentrations are significantly associated with eGFR (hs-cTnT: R:-0.396; hs-cTnI: R:-0.251; S2 Fig), regardless of the algorithm that is used to estimate GFR (data not shown). Also, both hs-cTnT and hs-cTnI concentrations are significantly correlated with CCS (hs-cTnT: R:0.279; hs-cTnI: R:0.213) and CCTA plaque severity (hs-cTnT: R:0.307; hs-cTnI, R:0.230) (Table 2, all p<0.001).

**Table 2:** Univariable and multivariable linear regression analysis, demonstrating the independent influence of eGFR on hs-cTn concentrations beyond CCTA parameters.

	Univariable models			Unadjusted multivariable model			Adjusted multivariable models								
							Model 1			Model 2			Model 3		
	St β	P-value	R²	St β	P-value	R²	St β	P-value	R²	St β	P-value	R²	St β	P-value	R²
*Dependent variable: Ln(hs-cTnT)*															
**eGFR**	**-0.396**	**<0.001**	0.156	**-0.347**	**<0.001**	0.224	**-0.289**	**<0.001**	0.323			0.282	**-0.290**	**<0.001**	0.338
**Coronary calcium score**	**0.279**	**<0.001**	0.077	**0.140**	**<0.001**					**0.078**	**0.002**		**0.089**	**<0.001**	
**CCTA plaque severity score**	**0.307**	**<0.001**	0.094	**0.158**	**<0.001**					**0.074**	**0.004**		**0.062**	**0.012**	
Age							0.254	<0.001		0.357	<0.001		0.201	<0.001	
Female sex							-0.336	<0.001		-0.278	<0.001		-0.307	<0.001	
BMI							0.007	0.726		0.047	0.022		0.009	0.659	
Diabetes							0.066	0.001		0.056	0.006		0.061	0.002	
Family history							-0.025	0.197		-0.046	0.023		-0.033	0.085	
Systolic blood pressure							0.057	0.014		0.052	0.031		0.055	0.017	
Diastolic blood pressure							-0.028	0.224		-0.030	0.206		-0.033	0.151	
Total cholesterol							-0.042	0.032		-0.043	0.035		-0.036	0.069	
*Dependent variable: Ln(hs-cTnI)*															
**eGFR**	**-0.251**	**<0.001**	0.063	**-0.212**	**<0.001**	0.104	**-0.222**	**<0.001**	0.145			0.129	**-0.222**	**<0.001**	0.159
**Coronary calcium score**	**0.213**	**<0.001**	0.045	**0.113**	**<0.001**					**0.092**	**<0.001**		**0.101**	**<0.001**	
**CCTA plaque score**	**0.230**	**<0.001**	0.052	**0.122**	**<0.001**					**0.072**	**0.011**		**0.062**	**0.026**	
Age							0.095	0.001		0.158	<0.001		0.038	0.186	
Female sex							-0.261	<0.001		-0.207	<0.001		-0.231	<0.001	
BMI							0.031	0.174		0.061	0.006		0.032	0.145	
Diabetes							-0.022	0.319		-0.032	0.152		-0.028	0.202	
Family history							-0.013	0.544		-0.032	0.158		-0.022	0.317	
Systolic blood pressure							0.098	<0.001		0.094	<0.001		0.096	<0.001	
Diastolic blood pressure							-0.017	0.522		-0.019	0.467		-0.022	0.400	
Total cholesterol							0.018	0.435		0.018	0.431		0.025	0.266	

**Fig 2:**
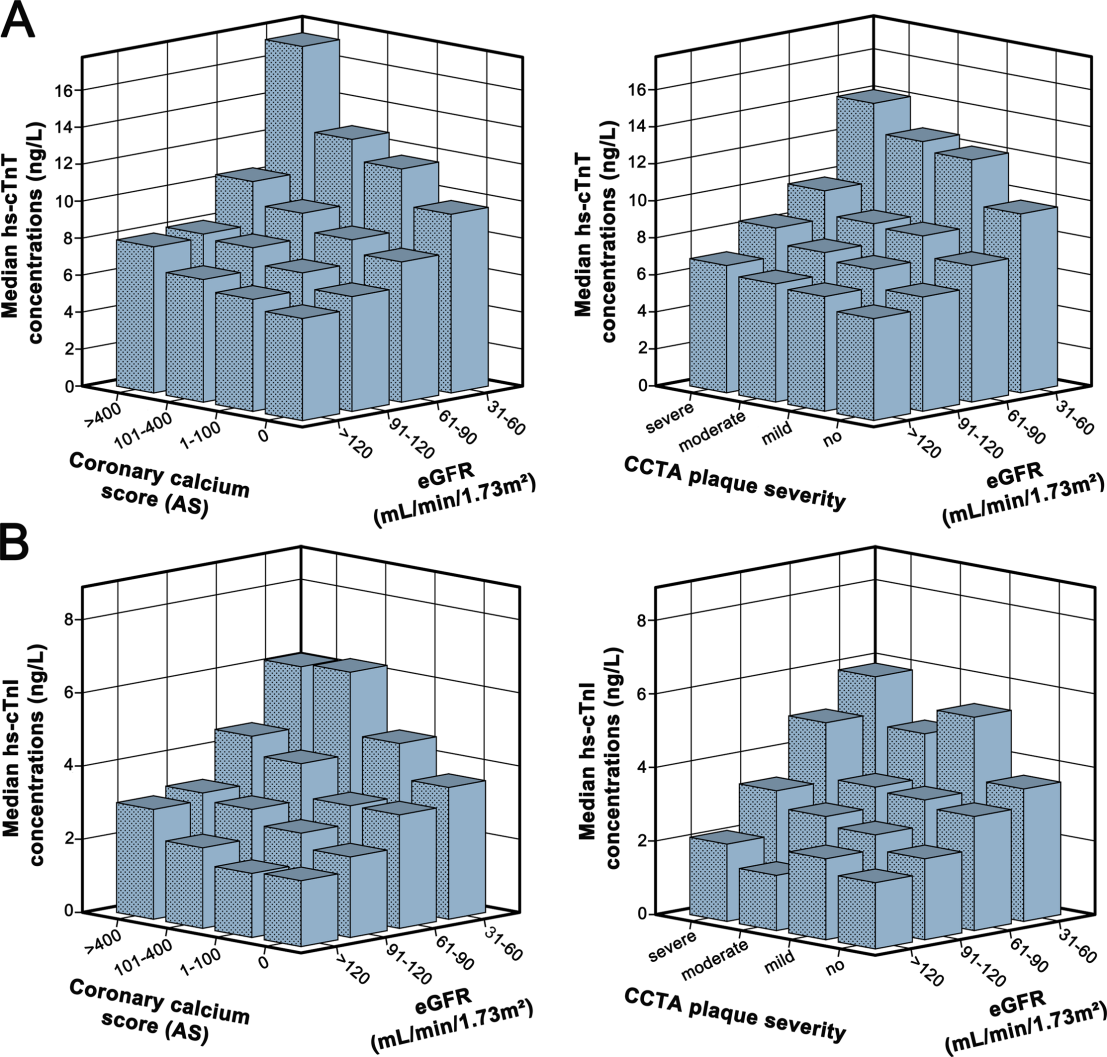
**Median hs-cTnT (A) and hs-cTnI (B) concentrations** according to estimated GFR categories and coronary calcium score or CCTA plaque severity. *AS*, *agatston score*.

As displayed by Fig 2, when adding renal and CT parameters as explanatory variables for hs-cTn concentrations in a multivariable regression model, both eGFR, CCS as CCTA plaque severity were identified as independent predictors (Table 2, unadjusted multivariable model).

Similar standardized β_eGFR_ (stβ_eGFR_) coefficients were observed in the univariable versus unadjusted multivariable models, indicating that CT parameters and eGFR hardly influenced each other when predicting hs-cTnT or hs-cTnI concentrations (Table 2).

After adjustment for traditional cardiovascular risk factors, eGFR, CCS and CCTA plaque severity score remained significantly associated with hs-cTn concentrations (Table 2, adjusted multivariable model 1 and 2, respectively). Also here, stβ_eGFR,_ stβ_CCS_ and stβ_CCTA_ values remain unchanged (Table 2, adjusted multivariable models 1–3).The independent contribution of eGFR to the prediction of hs-cTnT and hs-cTnI concentrations was also demonstrated by significant and identical R² changes (hs-cTnT: 0.056, hs-cTnI: 0.030; all p<0.001) when adding eGFR either to the baseline model or to adjusted multivariable model 2.

Furthermore, the association of eGFR with hs-cTn concentrations remained equally strong when subdividing this cohort into patients with no (hs-cTnT:stβ_eGFR_:-0.295; hs-cTnI:stβ_eGFR_:-0.228), mild (hs-cTnT:stβ_eGFR_:-0.290; hs-cTnI:stβ_eGFR_:-0.176) and moderate-to-severe CAD (hs-cTnT:stβ_eGFR_:-0.293;hs-cTnI: stβ_eGFR_:-0.249) (all p<0.001), confirming the independent influence of eGFR on hs-cTn concentrations beyond CAD severity (S1 Table). This finding was visible but less apparent for hs-cTnI than for hs-cTnT.

In a subgroup of this cohort, also echocardiographic parameters were included as explanatory variables for hs-cTn concentrations. Univariably, hs-cTnT and hs-cTnI were significantly associated with the echocardiographic measures LVEF (hs-cTnT: R:-0.151, p = 0.001; hs-cTnI, R:-0.142, p = 0.002) and LVmass (hs-cTnT: R:0.253; hs-cTnI: R:0.309; p<0.001) (S2 Table). In line with previous results, stβ_eGFR_ coefficients were only influenced by the confounding effects of traditional cardiovascular risk factors and not by any of the measured CT parameters or echocardiographic parameters (Table 3).

**Table 3:** Adjusted multivariable linear regression analysis for the influence of CT and echocardiographic parameters and eGFR on hs-cTn concentrations (N = 549/1864). Almost identical standardized β values (stβ) for eGFR were observed when comparing adjusted multivariable models 1, 3 and 5, indicating the independent influence of eGFR on hs-cTn concentrations beyond echocardiographic parameters.

	Adjusted multivariable models														
	Model 1			Model 2			Model 3			Model 4			Model 5		
	St β	P-value	R²	St β	P-value	R²	St β	P-value	R²	St β	P-value	R²	St β	P-value	R²
*Dependent variable: LN(hs-cTnT)*															
**eGFR**	**-0.244**	**<0.001**	0.261			0.241	**-0.247**	**<0.001**	0.282			0.256	**-0.254**	**<0.001**	0.298
**LVmass**				**0.132**	**0.004**		**0.136**	**0.003**		**0.139**	**0.002**		**0.144**	**0.001**	
**LVEF**				**-0.074**	**0.077**		**-0.075**	**0.065**		**-0.065**	**0.115**		**-0.066**	**0.101**	
**Coronary calcium score**										**0.132**	**0.002**		**0.142**	**0.001**	
Age	0.255	<0.001		0.378	<0.001		0.245	<0.001		0.335	<0.001		0.195	<0.001	
Gender	-0.288	<0.001		-0.212	<0.001		-0.228	<0.001		-0.193	<0.001		-0.208	<0.001	
BMI	-0.016	0.700		-0.018	0.676		-0.048	0.247		-0.017	0.687		-0.048	0.242	
Diabetes	0.076	0.064		0.062	0.135		0.067	0.099		0.064	0.118		0.069	0.083	
Family history	-0.040	0.312		-0.037	0.357		-0.025	0.534		-0.037	0.359		-0.024	0.543	
Systolic BP	0.041	0.396		0.026	0.593		0.022	0.639		0.029	0.542		0.026	0.582	
Diastolic BP	-0.060	0.200		-0.046	0.337		-0.051	0.271		-0.055	0.240		-0.062	0.179	
Total cholesterol	-0.079	0.053		-0.072	0.081		-0.069	0.086		-0.066	0.109		-0.062	0.118	
*Dependent variable: LN(hs-cTnI)*															
**eGFR**	**-0.207**	**<0.001**	0.120			0.141	**-0.213**	**<0.001**	0.171			0.160	**-0.220**	**<0.001**	0.191
**LVmass**				**0.225**	**<0.001**		**0.229**	**<0.001**		**0.234**	**<0.001**		**0.238**	**<0.001**	
**LVEF**				**-0.080**	**0.071**		**-0.081**	**0.063**		**-0.070**	**0.109**		**-0.071**	**0.100**	
**Coronary calcium score**										**0.150**	**0.001**		**0.157**	**<0.001**	
Age	0.085	0.008		0.185	<0.001		0.068	0.199		0.136	0.005		0.014	0.804	
Gender	-0.253	<0.001		-0.141	0.003		-0.155	0.001		-0.120	0.012		-0.134	0.005	
BMI	0.010	0.821		-0.020	0.654		-0.046	0.308		-0.019	0.669		-0.045	0.307	
Diabetes	-0.019	0.674		-0.032	0.465		-0.028	0.517		-0.030	0.496		-0.025	0.555	
Family history	-0.019	0.656		-0.009	0.834		0.001	0.978		-0.009	0.838		0.002	0.965	
Systolic BP	0.110	0.037		0.081	0.123		0.080	0.121		0.084	0.105		0.083	0.102	
Diastolic BP	-0.071	0.171		-0.047	0.356		-0.055	0.276		-0.058	0.252		-0.066	0.183	
Total cholesterol	-0.031	0.489		-0.020	0.649		-0.017	0.691		-0.013	0.765		-0.010	0.820	

In total, 30% and 19% of hs-cTnT and hs-cTnI variation, respectively, could be explained by the most important independent predictors: eGFR, CCS, LVmass, age and male sex (Table 3).

### Hs-cTn remain significant and comparable prognostic markers for adverse events

Over a mean follow-up period of 2.9±1.5 years, 68 adverse events (4.0%) were observed, encompassing 33 patients who underwent late revascularization, 18 patients that suffered from a non-fatal acute coronary syndrome and 17 patients who died. From all traditional risk factors, only age, smoking and total cholesterol were significantly different in the event versus non-event group (S3 Table).

As depicted in Table 4, univariable Cox regression analysis showed that the incidence of adverse events was significantly associated with hs-cTnT and hs-cTnI. Both hs-cTn results remained highly significant predictors for adverse events when adjusted for significant traditional risk factors and even CCS (Table 4) and CAD-severity (S4 Table). Overall, patients with elevated hs-cTnT (>14 ng/L) and hs-cTnI (>26.2 ng/L) concentrations were respectively 3 and 8 times more at risk for adverse events on follow-up.

**Table 4:** Cox proportional regression analysis for the association of hs-cTnT and hs-cTnI with adverse events in all patients or when stratified for eGFR ≥or <90mL/min/1.73m².

	All patients				eGFR≥90mL/min/1.73m^2^				eGFR<90mL/min/1.73m^2^			
	(n = 1689, 68 events)				(n = 1192, 34 events)				(n = 497, 34 events)			
	univariable		Multivariable*		univariable		Multivariable*		univariable		Multivariable*	
	HR (95%CI)	P-value	HR (95%CI)	P-value	HR (95%CI)	P-value	HR (95%CI)	P-value	HR (95%CI)	P-value	HR (95%CI)	P-value
**Hs-cTnT**	1.025	<0.001	1.028	<0.001	1.018	0.020	1.019	0.031	1.071	<0.001	1.076	<0.001
**(ng/L)**	*(1*.*016–1*.*033)*		*(1*.*017–1*.*039)*		*(1*.*003–1*.*034)*		*(1*.*002–1*.*037)*		*(1*.*048–1*.*094)*		*(1*.*043–1*.*111)*	
**Hs-cTnT**	4.806	<0.001	2.804	0.001	3.289	0.025	2.321	0.135	4.631	<0.001	2.909	0.010
**>99**^**th**^ **perc**	*(2*.*698–8*.*561)*		*(1*.*495–5*.*260)*		*(1*.*158–9*.*342)*		*(0*.*770–7*.*00)*		*(2*.*217–9*.*673)*		*(1*.*287–6*.*572)*	
**(14 ng/L)**												
**Hs-cTnI**	1.007	<0.001	1.008	<0.001	1.006	0.002	1.007	0.001	1.015	<0.001	1.014	0.001
**(ng/L)**	*(1*.*004–1*.*010)*		*(1*.*005–1*.*012)*		*(1*.*002–1*.*010)*		*(1*.*003–1*.*011)*		*(1*.*007–1*.*022)*		*(1*.*005–1*.*024)*	
**Hs-cTnI**	10.250	<0.001	7.768	<0.001	9.529	<0.001	8.239	<0.001	10.875	<0.001	11.147	<0.001
**>99**^**th**^ **perc**	*(5*.*218–20*.*13)*		*(3*.*814–15*.*82)*		*(3*.*685–24*.*64)*		*(3*.*012–22*.*54)*		*(4*.*162–28*.*42)*		*(3*.*868–32*.*12)*	
**(26.2 ng/L)**												

*multivariable models are also adjusted to age, gender, smoking, total cholesterol and CCS

However, renal function significantly affected the association between hs-cTnT and hs-cTnI with adverse events (p-value interaction term, <0.001 and 0.007, respectively). Higher hs-cTnT concentrations showed lower survival rates for adverse cardiac events when eGFR was <90 mL/min/1.73m² in comparison when eGFR was ≥90 mL/min/1.73m² (as illustrated by the Kaplan-Meier curves in S3 Fig). Consequently, in patients with eGFR <90mL/min/1.73m², a 1 ng/L-rise in hs-cTnT and hs-cTnI resulted in an increased relative risk of 7.1% and 1.5% respectively. However this increase in relative risk was 4 to 2 times less for hs-cTnT (2%) and hs-cTnI (0.6%) in patients with normal renal function (eGFR ≥90 mL/min/1.73m²).

## Discussion

The present study provides new insights into the interpretation of hs-cTn concentrations in patients with chest discomfort, identifying not only cardiac parameters but also renal function as independent and strong contributors to circulating hs-cTn concentrations. In fact, eGFR exhibited limited confounding effects on the association between hs-cTn and stable CAD but did interfere with the association between hs-cTn and the risk on adverse events, such as mortality and AMI.

### Influence of renal function on hs-cTnT and hs-cTnI concentrations

In patients with chest discomfort, we found that hs-cTnT and hs-cTnI were strongly correlated with eGFR. Also in patients with noncardiac cause of chest pain, eGFR was found to be next to age an important determinant for hs-cTnT concentrations [29]. Even within normal eGFR boundaries, our results clearly demonstrate that a decreased renal clearance affects is associated with hs-cTn concentrations. Although differences were small, hs-cTnT was more strongly correlated with eGFR than hs-cTnI, which was also reported in subjects with moderate-to-severe CKD [13,30]. In addition, the association between hs-cTnT concentrations and eGFR were stronger than any known associations between hs-cTnT and cardiac parameters, such as coronary plaque severity [2,3,31] or left ventricular structure [16,18,32]. Interestingly, this is in contrast to hs-cTnI, in which the association with LVmass was greater than with eGFR. For clinical decision making, hs-cTnT and hs-cTnI assays are currently used interchangeably from each other. However, these findings suggest that hs-cTnT could be more susceptible to changes in renal clearance than hs-cTnI. Future research should further examine these differences between hs-cTnT and hs-cTnI.

### Renal and cardiac parameters are two independent contributors to hs-cTn

Importantly, we demonstrate for the first time that the extent of stable CAD and echocardiographic characteristics of the left ventricle did not interfere with the magnitude of the association between eGFR and hs-cTn concentrations. As a consequence, in non-acute patients with a normal to mildly impaired kidney function, decreases in renal function and the extent of stable CAD can be considered as two contributors of hs-cTn accumulation. Further research is necessary to examine the association of hs-cTn with renal function relative to cardiovascular disease in populations such as acute chest pain and chronic heart failure patients.

The significant association between eGFR and hs-cTn concentrations, independent from cardiac pathologies, is in line with the observation that cTnT and cTnI are cleared by the kidneys [33,34]. However, we cannot exclude that a decreased renal function may exert additional myocardial stress that is not identified by cardiac imaging, leading to subsequent cardiac troponin release. We were able to explain up to 30% of the variation in hs-cTn concentrations, and therefore unknown or undetected pathologies to the myocardium can also result in cTn release that was not taken into account.

Furthermore, these findings provide an explanation for the reduced diagnostic performance of hs-cTn seen in AMI patients with lower renal function in comparison to those with normal renal function [35,36]. Therefore, when acquiring the diagnostic hs-cTn cutoffs from reference populations (99^th^ upper reference limit), it is of equal importance to screen for renal as for cardiac health.

### Significant influence of renal function on the prognostic value of hs-cTn

In this study, we found that serum hs-cTnT as well as hs-cTnI were significant prognostic markers for the prediction of adverse events, independent from other established risk predictors, such as CCS. These results therefore indicate that both hs-cTn are not only useful risk stratifyers in patients at serious risk for adverse events [8,9,32,37], but also contribute significantly in a low-risk stable CAD population.

When adjusted for decreased renal function, hs-cTnT and hs-cTnI remain significant prognostic markers for adverse events, as found in other study populations [38,39], although renal function significantly modified this association. Therefore, increases in hs-cTnT and hs-cTnI indicate respectively 4 and 2 times more risk in patients with reduced renal function compared to patients with normal renal function. The rationale behind these results could be in line with previous findings that patients with decreased renal function are more at risk for developing events [40,41]. Moreover, the attenuation in hazard ratios was more pronounced for hs-cTnT than hs-cTnI, and correspond to our previous observation that hs-cTnT was more associated with renal function than hs-cTnI. Nonetheless, in the patients with both reduced and normal eGFR, hs-cTnT and hs-cTnI concentrations hold an important prognostic value besides important risk predictors such as CCS, age and smoking. Prospective studies must establish whether baseline hs-cTn concentrations should therefore be accompanied by GFR-assessment.

## Conclusion

In conclusion, we identified in patients with symptoms of chest discomfort that renal function has a moderate and independent influence on circulating hs-cTnT and hs-cTnI concentrations. Moreover, renal function significantly affected the association of hs-cTnT and hs-cTnI with adverse events. Therefore, our results seriously question whether baseline hs-cTn concentrations should be reported without the access to an eGFR.

## Supporting Information

S1 Fig Scatterplots and simple linear regression of the association between Ln(hs-cTnT) and Ln(hs-cTnI). The regression line follows the linear function y = 1.37 x—1.75.
(DOCX)

S2 Fig Scatterplots of the association of eGFR with Ln (hs-cTnT) (A) and Ln (hs-cTnI) (B).
(DOCX)

S3 Fig Kaplan-Meier curves for the estimation of risk on all-cause mortality according to different eGFR and hs-cTnT or hs-cTnI categories. hs-cTnT_high_ indicates hs-cTnT > 4th quartile (= 9.2 ng/L); hs-cTnTl_ow_, hs-cTnT <4th quartile; hs-cTnI_high_, hs-cTnI >4th quartile (4.1 ng/L); hs-cTnI_low_, hs-cTnI <4th quartile; eGFR_normal_ indicates eGFR >90 mL/min/1.73m^2^; eGFR_reduced_ eGFR, <90 mL/min/1.73 m^2^.
(DOCX)

S1 Table Association of hs-cTnT or hs-cTnI concentrations with (A) eGFR in patients without CAD (n = 756), mild (n = 667) and moderate-to-severe CAD (n = 441) and (B) CCS in patients with eGFR < 90 mL/min/1.73m² (n = 524) and eGFR > = 90 mL/min/1.73m² (n = 1340), indicating almost identical unstandardized and standardized β values for eGFR (stβ_eGFR_) and CCS (stβ_CCS_) across these different categories. *, indicates Stβ obtained from the regression model containing also the variables age, gender, BMI, Family history, Systolic BP, diastolic BP and total cholesterol; †, p<0.001.
(DOCX)

S2 Table Baseline characteristics of subpopulation (N = 549/1876 patients) in which echocardiography was performed, by median hs-cTnT and hs-cTnI concentrations. (DOCX)

S3 Table Differences in baseline characteristics between cardiovascular event and event-free group. (DOCX)

S4 Table Cox proportional regression analysis for the association of hs-cTnT and hs-cTnI with adverse events in all patients or when stratified for eGFR ≥or <90mL/min/1.73m². All models were adjusted to age, gender, smoking, total cholesterol and CAD-severity score.
(DOCX)
