# Supplementary material for: High-Sensitivity Cardiac Troponin Concentrations in Patients with Chest Discomfort: Is It the Heart or the Kidneys As Well?
Source: PLoS One. 2016 Apr 20;11(4):e0153300. doi: 10.1371/journal.pone.0153300 (PMC4838230; doi:10.1371/journal.pone.0153300)
Supplement: S1 Fig — The regression line follows the linear function y = 1.37 x—1.75. (DOCX) [file pone.0153300.s001.docx]

**S1 Fig. Scatterplots and simple linear regression of the association between Ln(hs-cTnT) and Ln(hs-cTnI).** The regression line follows the linear function y= 1.37 x - 1.75

**
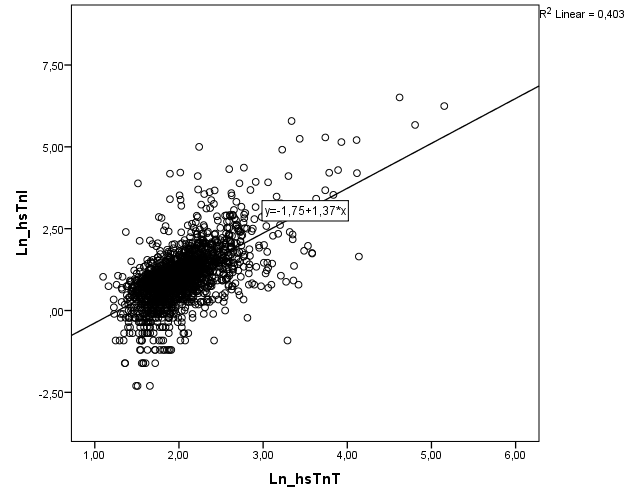
**
