# Supplementary figures and images for: High-Sensitivity Cardiac Troponin Concentrations in Patients with Chest Discomfort: Is It the Heart or the Kidneys As Well?
Source: PLoS One. 2016 Apr 20;11(4):e0153300. doi: 10.1371/journal.pone.0153300 (PMC4838230; doi:10.1371/journal.pone.0153300)

**S2 Fig. Scatterplots of the association of eGFR with Ln (hs-cTnT) (A) and Ln (hs-cTnI) (B).**

**
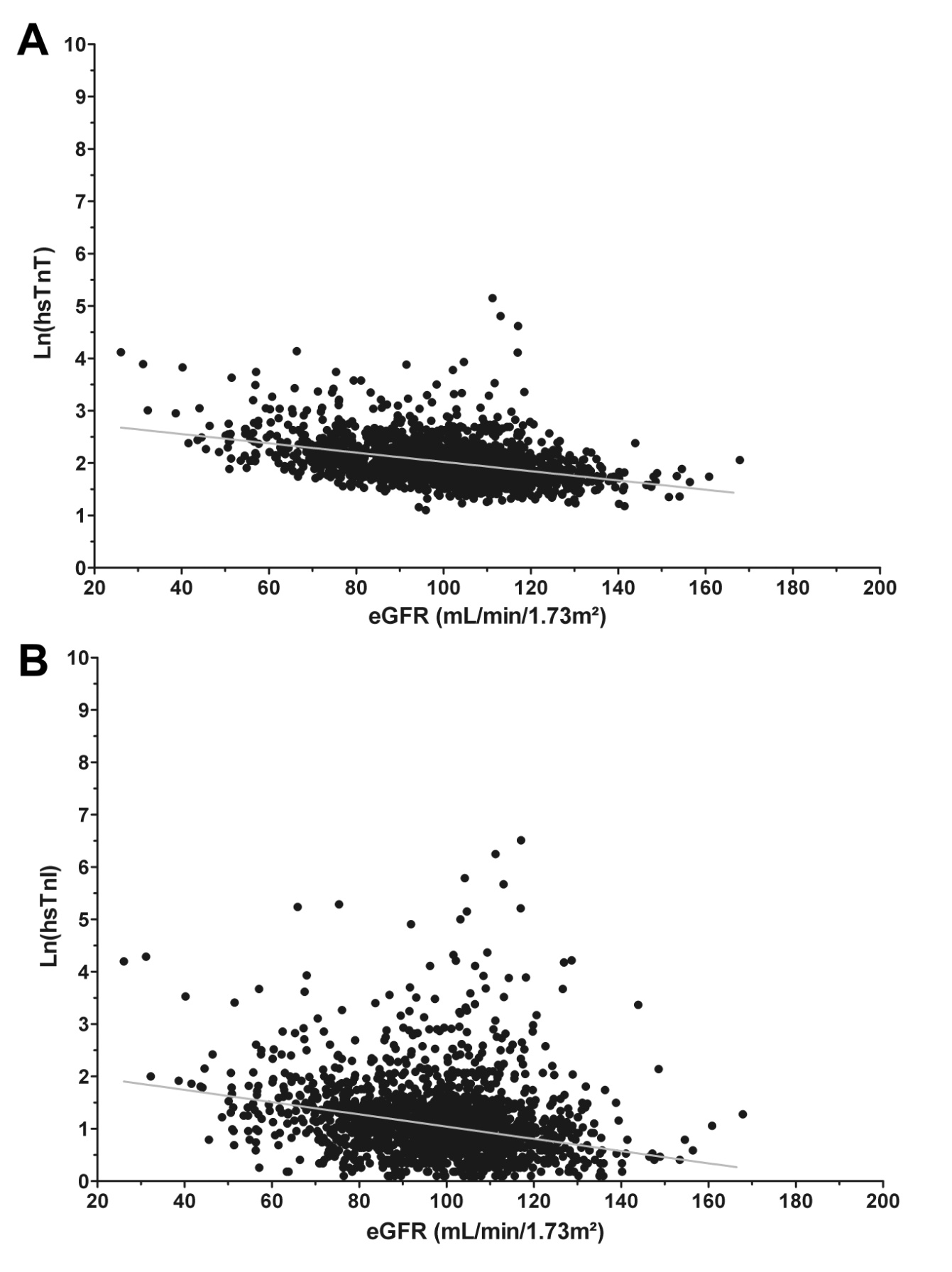
**

Supplement: S2 Fig — Scatterplots of the association of eGFR with Ln (hs-cTnT) (A) and Ln (hs-cTnI) (B). (DOCX) [file pone.0153300.s002.docx]
