# Supplementary material for: High-Sensitivity Cardiac Troponin Concentrations in Patients with Chest Discomfort: Is It the Heart or the Kidneys As Well?
Source: PLoS One. 2016 Apr 20;11(4):e0153300. doi: 10.1371/journal.pone.0153300 (PMC4838230; doi:10.1371/journal.pone.0153300)
Supplement: S3 Fig — hs-cTnThigh indicates hs-cTnT > 4th quartile (= 9.2 ng/L); hs-cTnTlow, hs-cTnT <4th quartile; hs-cTnIhigh, hs-cTnI >4th quartile (4.1 ng/L); hs-cTnIlow, hs-cTnI <4th quartile; eGFRnormal indicates eGFR >90 mL/min/1.73m2; eGFRreduced eGFR, <90 mL/min/1.73 m2. (DOCX) [file pone.0153300.s003.docx]

**S3 Fig. Kaplan-Meier curves for the estimation of risk on all-cause mortality according to different eGFR and hs-cTnT or hs-cTnI categories.** hs-cTnT_high_ indicates hs-cTnT > 4th quartile (= 9.2 ng/L); hs-cTnTl_ow_, hs-cTnT <4th quartile; hs-cTnI_high_, hs-cTnI >4th quartile (4.1 ng/L); hs-cTnI_low_, hs-cTnI <4th quartile; eGFR_normal_ indicates eGFR >90 mL/min/1.73m^2^; eGFR_reduced_ eGFR, <90 mL/min/1.73 m^2^.

**

**
