# Supplementary material for: High-Sensitivity Cardiac Troponin Concentrations in Patients with Chest Discomfort: Is It the Heart or the Kidneys As Well?
Source: PLoS One. 2016 Apr 20;11(4):e0153300. doi: 10.1371/journal.pone.0153300 (PMC4838230; doi:10.1371/journal.pone.0153300)
Supplement: S1 Table — *, indicates Stβ obtained from the regression model containing also the variables age, gender, BMI, Family history, Systolic BP, diastolic BP and total cholesterol; †, p<0.001. (DOCX) [file pone.0153300.s004.docx]

**S1 Table.** **Association of hs-cTnT or hs-cTnI concentrations with (A) eGFR in patients without CAD (n= 756), mild (n= 667) and moderate-to-severe CAD (n= 441) and (B) CCS in patients with eGFR < 90 mL/min/1.73m² (n=524) and eGFR >= 90 mL/min/1.73m² (n=1340) ,** indicating almost identical unstandardized and standardized β values for eGFR (stβ_eGFR_) and CCS (stβ_CCS_) across these different categories. *, indicates Stβ obtained from the regression model containing also the variables age, gender, BMI, Family history, Systolic BP, diastolic BP and total cholesterol; †, p<0.001

|  | | *Dependent variable: Ln(hs-cTnT)* | | *Dependent variable: Ln(hs-cTnI)* | |
| --- | --- | --- | --- | --- | --- |
| ***A. CCTA plaque severity*** | | **St β_eGFR_(95%CI)*** | **R²** | **St β_eGFR_(95%CI)*** | **R²** |
| **No CAD** | eGFR | -0.295  (-0.373;-0.218)† | 0.237 | -0.228  (-0.313;-0.144)† | 0.107 |
| **Mild CAD** | eGFR | -0.290  (-0.368;-0.213)† | 0.297 | -0.176  (-0.264;-0.088)† | 0.094 |
| **Moderate-to-severe CAD** | eGFR | -0.293  (-0.386;-0.200)† | 0.275 | -0.249  (-0.350;-0.148)† | 0.151 |
| ***B. eGFR category*** |  | **St β_eGFR_(95%CI)*** | **R²** | **St β_eGFR_(95%CI)*** | **R²** |
| **eGFR < 90 mL/min/1.73m²** | CCS | 0.121  (0.043-0.199) | 0.287 | 0.119  (0.032-0.206) | 0.121 |
| **eGFR >= 90 mL/min/1.73m²** | CCS | 0.123  (0.072-0.174 | 0.226 | 0.140  (0.085-0.195) | 0.109 |
