# Supplementary material for: High-Sensitivity Cardiac Troponin Concentrations in Patients with Chest Discomfort: Is It the Heart or the Kidneys As Well?
Source: PLoS One. 2016 Apr 20;11(4):e0153300. doi: 10.1371/journal.pone.0153300 (PMC4838230; doi:10.1371/journal.pone.0153300)
Supplement: S2 Table — (DOCX) [file pone.0153300.s005.docx]

**S2 Table.** **Baseline characteristics of subpopulation** (N=549/1876 patients) **in which echocardiography was performed**, by median hs-cTnT and hs-cTnI concentrations.

|  |  | **hs-cTnT** | |  | **hs-cTnI** | | |  | |
| --- | --- | --- | --- | --- | --- | --- | --- | --- | --- |
| **Determinant** | **All patients** | **≤7.2 ng/L**  **(N=231)** | **>7.2 ng/L**  **(N=318)** | **P-value** | | **≤2.6 ng/L**  **(N=163)** | **>2.6 ng/L**  **(N=386)** | **P-value** | |
| **Echocardiography** |  | |  |  | |  |  |  |  |
| Left ventricular ejection fraction,% | 60.0±7.3 | 60.7±5.9 | 59.4±8.2 | 0.037 | | 60.8±5.5 | 59.6±8.0 | 0.056 | |
| Left ventricular mass, g | 169.3±45.0 | 158.0±37.0 | 178.0±48.6 | <0.001 | | 155.0±33.6 | 175.9±48.0 | <0.001 | |
| Left ventricular mass index, g/m² | 87.4±20.0 | 83.2±16.6 | 90.7±21.8 | <0.001 | | 82.5±16.1 | 89.7±21.2 | <0.001 | |
|  | | | | | | | | | |
