# Supplementary material for: High-Sensitivity Cardiac Troponin Concentrations in Patients with Chest Discomfort: Is It the Heart or the Kidneys As Well?
Source: PLoS One. 2016 Apr 20;11(4):e0153300. doi: 10.1371/journal.pone.0153300 (PMC4838230; doi:10.1371/journal.pone.0153300)
Supplement: S3 Table — (DOCX) [file pone.0153300.s006.docx]

**S3 Table.** **Differences in baseline characteristics between the event and event-free group.**

| **Determinant** | **Event (N=68)** | **No event (N=1621)** | **P-value** |
| --- | --- | --- | --- |
| **Traditional risk factors** | | | |
| Males | 44 (64.7%) | 899 (55.5%) | 0.137 |
| Age, years | 61.4 ± 11.7 | 56.1 ± 10.7 | <0.001 |
| BMI, kg/m² | 27.0 ± 5.0 | 27.0 ± 4.3 | 0.913 |
| Smokers | 39.7% | 21.7% | 0.001 |
| Diabetes | 7 (10.3%) | 121 (7.5%) | 0.351 |
| Fam. history | 23 (33.8%) | 621 (38.3%) | 0.525 |
| Systolic BP, mmHg | 143.8 ± 17.8 | 142.6 ± 19.5 | 0.591 |
| Diastolic BP, mmHg | 78.7 ± 13.0 | 80.1 ± 11.4 | 0.287 |
| Total cholesterol, mmol/L | 5.1 ± 1.4 | 5.4 ± 1.2 | 0.040 |
| **Cardiac biochemical markers** | | | |
| hs-cTnT, ng/L | 9.0 (7.0-13.8) | 7.2 (5.8-9.1) | 0.002 |
| hs-cTnI, ng/L | 3.7 (2.4-10.1) | 2.7 (1.8-4.1) | 0.021 |
| **Renal clearance** | | | |
| Cystatin C, mg/L | 0.89 ± 0.29 | 0.77 ± 0.17 | 0.001 |
| Creatinine, µmol/L | 80.8 ± 22.2 | 74.8 ± 15.6 | 0.031 |
| eGFR_creat+cysC_, mL/min/1.73m² | 89.3 ± 22.1 | 99.2 ± 18.1 | <0.001 |
| **CT/CCTA parameters** | | | |
| Coronary calcium score, AS | 175.4 (8.3-556.7) | 4.3 (0-102) | <0.001 |
| Moderate-to-severe plaque | 44 (64.7%) | 371 (22.9%) | <0.001 |

*Data is indicated as either: n (%), mean (±SD) or median (IQR)*
