# Supplementary material for: High-Sensitivity Cardiac Troponin Concentrations in Patients with Chest Discomfort: Is It the Heart or the Kidneys As Well?
Source: PLoS One. 2016 Apr 20;11(4):e0153300. doi: 10.1371/journal.pone.0153300 (PMC4838230; doi:10.1371/journal.pone.0153300)
Supplement: S4 Table — All models were adjusted to age, gender, smoking, total cholesterol and CAD-severity score. (DOCX) [file pone.0153300.s007.docx]

**S4 Table. Cox proportional regression analysis for the association of hs-cTnT and hs-cTnI with adverse events in all patients or when stratified for eGFR ≥or <90mL/min/1.73m².**  *All models were adjusted to age, gender, smoking, total cholesterol and CAD-severity score*

|  | **All patients**  **(n=1689, 68 events)** | | **eGFR≥90mL/min/1.73m^2^**  **(n=1192, 34 events)** | | **eGFR<90mL/min/1.73m^2^**  **(n=497, 34 events)** | |
| --- | --- | --- | --- | --- | --- | --- |
|  | HR  (95%CI) | P-value | HR  (95%CI) | P-value | HR  (95%CI) | P-value |
| **Hs-cTnT** | 1.060  *(1.034-1.086)* | <0.001 | 1.020  *(1.003-1.037)* | 0.021 | 1.071  *(1.040-1.104)* | <0.001 |
| **Hs-cTnT**  **>99^th^ perc** | 3.133  *(1.666-5.890)* | <0.001 | 2.886  *(0.996-8.361)* | 0.051 | 2.959  *(1.307-6.698)* | 0.009 |
| **Hs-cTnI** | 1.008  *(1.005-1.012)* | <0.001 | 1.007  *(1.003-1.011)* | 0.001 | 1.012  *(1.003-1.021)* | 0.007 |
| **Hs-cTnI**  **>99^th^ perc** | 8.528  *(4.259-17.08)* | <0.001 | 9.459  *(3.614-24.757)* | <0.001 | 8.734  *(3.114-24.49)* | <0.001 |
